# Epidural hematoma in a pediatric patient with Hutchinson–Gilford progeria syndrome: management considerations: a case report

**DOI:** 10.1186/s41016-026-00431-z

**Published:** 2026-04-03

**Authors:** Ali Imran Ozmarasali, Zuhal Zeybek Sivas, Ilken Uguz, Arzu Oto

**Affiliations:** 1 Department of Neurosurgery, Bursa State Hospital, Bursa, 16110 Türkiye; 2 Department of Otorhinolaryngology-Head and Neck Surgery, Bursa State Hospital, Bursa, 16110 Türkiye; 3 Department of Anesthesiology, Bursa State Hospital, Bursa, 16110 Türkiye; 4 Division of Pediatric Critical Care, Department of Pediatrics, Bursa State Hospital, Bursa, 16110 Türkiye

**Keywords:** Hutchinson–Gilford Progeria Syndrome, Epidural Hematoma, Difficult Airway, Tracheostomy, Vascular Fragility, Neurosurgery

## Abstract

**Background:**

Hutchinson–Gilford progeria syndrome (HGPS) is an exceedingly rare genetic disorder characterized by accelerated aging and profound vascular fragility. While cerebrovascular accidents are common in this population, traumatic epidural hematomas (EDH) are clinical rarities, with only two pediatric cases previously documented. Currently, it is estimated that fewer than 200 children with HGPS are living worldwide.

The primary objective of this report is to present a comprehensive multidisciplinary management strategy for traumatic EDH in a 15-year-old HGPS patient, highlighting the critical interplay between progerin-induced vascular stiffening, reduced intracranial compliance, and the formidable anesthetic challenges encountered in the oldest reported survivor of this pathology.

**Case presentation:**

A 15-year-old female with HGPS presented with a right frontoparietal EDH following a ground-level fall. Initial Glasgow Coma Scale (GCS) was 14 but declined to 12 within 4 h. Cranial computed tomography (CT) revealed a large EDH measuring 22 mm at maximum thickness, causing a 6 mm midline shift. Due to severe glottic sclerosis and anatomical distortion, multiple orotracheal and fiberoptic (2.8 mm) intubation attempts failed. An emergency percutaneous tracheostomy was performed to secure the airway. Intraoperative findings revealed active bleeding from a coronal suture diastasis, which was managed with bone wax and dural tenting sutures. The patient achieved full neurological recovery and was discharged on postoperative day 10.

**Conclusion:**

Managing HGPS patients requires a high index of clinical suspicion for intracranial injury even after minor trauma, as progerin-induced vasculopathy accelerates hematoma expansion. Standard airway techniques can fail due to progressive laryngeal changes, necessitating early consideration of surgical airways. Prompt multidisciplinary intervention and meticulous neurosurgical technique are essential to mitigate risks associated with extreme vascular fragility and complex anatomy in this high-risk population.

## Background

Hutchinson–Gilford progeria syndrome (HGPS) is an exceedingly rare and fatal multisystemic genetic disorder characterized by dramatic, premature aging starting in early childhood. This condition is caused by a de novo point mutation in the LMNA gene, leading to the accumulation of an abnormal protein called progerin [1]. Progerin interferes with nuclear stability and cellular functions, resulting in progressive atherosclerosis, connective tissue atrophy, and skeletal abnormalities.

Patients with HGPS are highly susceptible to severe intracranial injuries even after low-energy impacts, primarily due to extreme vascular fragility [2, 3]. Despite the high risk of cerebrovascular events like strokes, traumatic epidural hematomas (EDH) are remarkably rare in this population. To date, only two cases of EDH in HGPS patients have been documented in the literature [4, 5].

The management of traumatic EDH in HGPS patients necessitates a sophisticated multidisciplinary approach, as the clinical picture is complicated by a unique intersection of pathophysiological factors. Progerin-induced vascular stiffening and accelerated medial calcification significantly compromise intracranial compliance and increase the risk of rapid neurological deterioration [2]. Simultaneously, characteristic craniofacial dysmorphisms—including severe micrognathia, limited atlanto-axial mobility, and progressive glottic sclerosis—present formidable obstacles to conventional airway management [6]. This synergy of extreme vascular fragility and high-risk anesthetic anatomy demands precise coordination between neurosurgical and anesthetic teams to ensure rapid decompression while mitigating perioperative morbidity.

The primary objective of this report is to present a comprehensive management strategy for traumatic EDH in a 15-year-old HGPS patient, who represents the oldest reported case with this pathology. We specifically highlight the interplay between extreme vascular fragility, the complexities of securing a difficult airway in an aging pediatric patient, and the necessity of urgent neurosurgical decompression in a high-risk physiological environment.

## Case presentation

### Clinical chronology and referral

A 15-year-old female patient with a known diagnosis of HGPS sustained a head injury following a ground-level fall at a playground. Two hours post-injury, she developed nausea and vomiting, prompting an evaluation at a secondary trauma center. Initial cranial computed tomography (CT) revealed a right frontoparietal epidural hematoma EDH, and her Glasgow Coma Scale (GCS) score was 14. Due to the high anesthetic risk and the anticipated difficulty of airway management in an HGPS patient, she was referred to our tertiary center for multidisciplinary management.

**Fig. 1:**
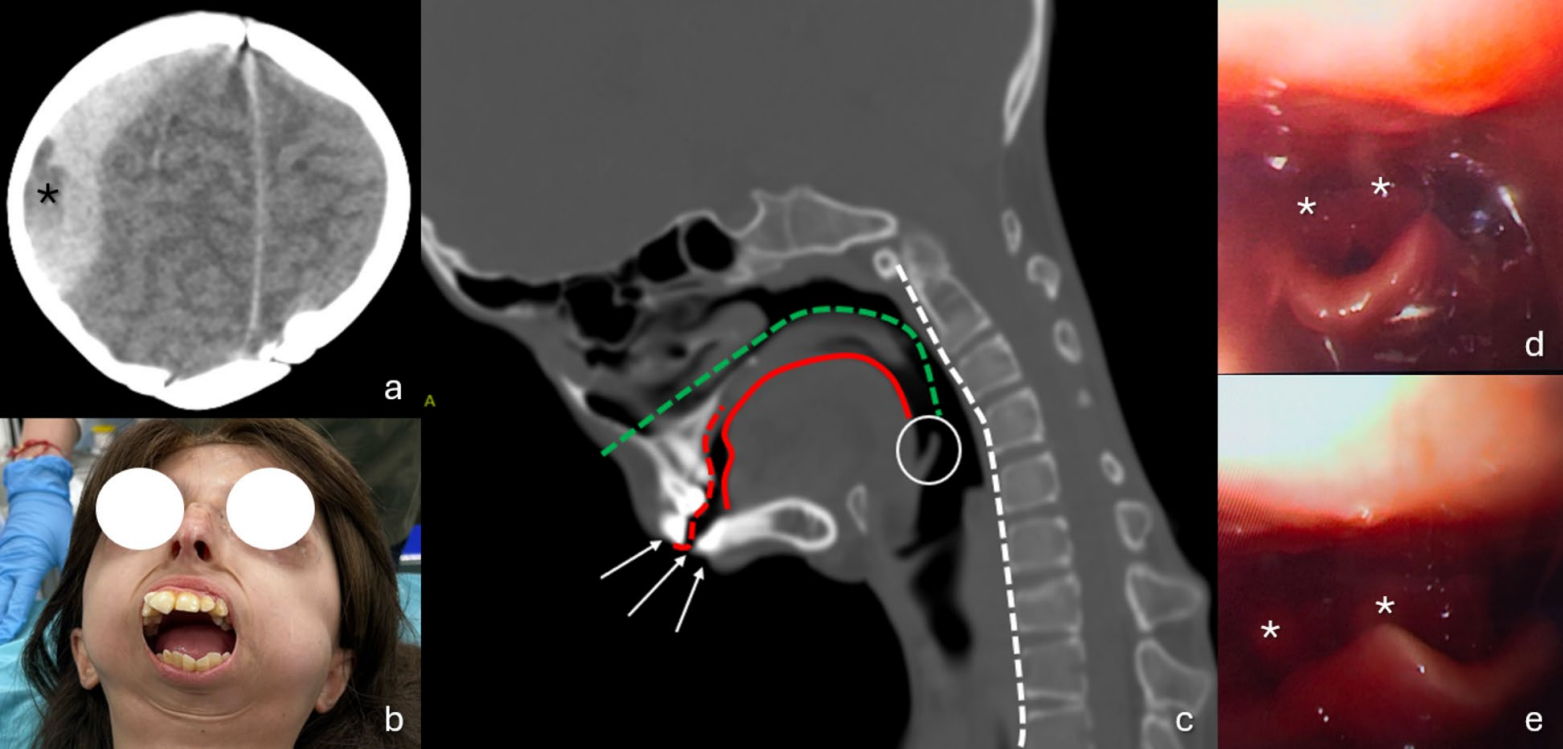
Preoperative axial noncontrast cranial CT (**a**), clinical photographs showing limited mouth opening and dentition (**b**), sagittal cervical CT (**c**), and endoscopic airway views (**d**–**e**). Preoperative evaluation revealed a swirl sign (**a**, black asterisk), a large right frontotemporoparietal epidural hematoma with midline shift, and a very low-lying glottis (**c**, white circle). Despite maximal oral opening (**b**), severe orthodontic abnormalities (**b** and **c**, red dashes), enlarged tongue volume occupying the oropharyngeal cavity (**c**, red curve), and conversion of ankylosis with cervical lordosis to kyphotic angulation (**c**, white dashes, Cobb angle 24.6°) rendered orotracheal intubation impossible (**b**, **c**). Although the glottis was visualized, passage of the intubation tube was obstructed due to glottic sclerosis, hypertrophic arytenoids (**d**, **e**, white asterisk), and limited neck mobility

During the 1.5-h inter-hospital transfer, the patient was stabilized with a pediatric cervical collar. Intravenous access was maintained, and maintenance fluids (0.9% saline) were administered with continuous monitoring of vital signs; no invasive airway intervention was required during transit. Upon arrival at our facility, her GCS had decreased to 12. CT revealed a large right frontoparietal epidural hematoma, measuring 22 mm at its maximum thickness, causing a 6 mm midline shift to the left (Fig. 1a). These findings, combined with the presence of a 'swirl sign' and the patient's rapidly deteriorating neurological status (GCS decline from 14 to 12), necessitated immediate surgical evacuation and the patient was taken to the operating room within 4 h of the initial trauma.

#### Preoperative assessment and patient profile

Her parents were cousins, and there was a family history of multiple deaths due to progeria. She had a healthy older sister and a sibling also diagnosed with progeria. Physical examination revealed severe cachexia (Height: 112 cm, Weight: 14 kg, Body Mass Index (BMI): 11.16 kg/m^2^) and characteristic craniofacial dysmorphisms, including micrognathia and cervical ankylosis. Vital signs on admission were stable (Blood Pressure: 105/65 mmHg, Heart Rate: 92 bpm, SpO^2^: 96% on room air), without evidence of the Cushing triad. Preoperative electrocardiography was within normal. Laboratory tests, including coagulation profiles (International Normalized Ratio (INR): 1.14, aPTT: 24.2 s), showed no evidence of bleeding diathesis.

#### Anesthetic management and airway challenges

To manage increased intracranial pressure (ICP), intravenous mannitol (0.5 g/kg) was administered, and the head was elevated to 30° with mild hyperventilation via face mask prior to induction. Anesthesia was induced with intravenous propofol (2 mg/kg) and fentanyl (1 mcg/kg). Neuromuscular blocking agents were intentionally avoided due to the patient’s severe cachexia and low muscle mass. Although cachexia is not a contraindication for neuromuscular blocking agents, they were intentionally avoided in this case to prevent potential prolonged paralysis and to maintain spontaneous respiration in the face of an anticipated difficult airway, where titrated dosing was considered but deferred for safety. Prior to administering any further agents, the adequacy of mask ventilation was assessed and confirmed. Invasive arterial blood pressure (IBP) monitoring was established via the radial artery to ensure continuous hemodynamic monitoring.

Airway management proved exceptionally challenging. Given the anticipated difficult airway, the team was prepared for advanced techniques; however, initial orotracheal intubation attempts using a size 2 Macintosh blade with 5.0 mm and 6.0 mm cuffed tubes failed due to severe glottic sclerosis and anatomical distortion (Fig. 1b, c). As part of our escalation plan, a 2.8 mm diameter fiberoptic bronchoscope was immediately utilized; however, visualization was obscured by hypertrophic arytenoids and a high-positioned epiglottis, preventing tube passage (Fig. 1d, e). Given the rapidly declining oxygen saturation and the urgency of the hematoma, an emergency percutaneous tracheostomy was performed using a 5.0 mm cannula. Maintenance of anesthesia was achieved with sevoflurane (2%) and a medical air/oxygen mixture.

#### Surgical ıntervention and postoperative course

**Fig. 2:**
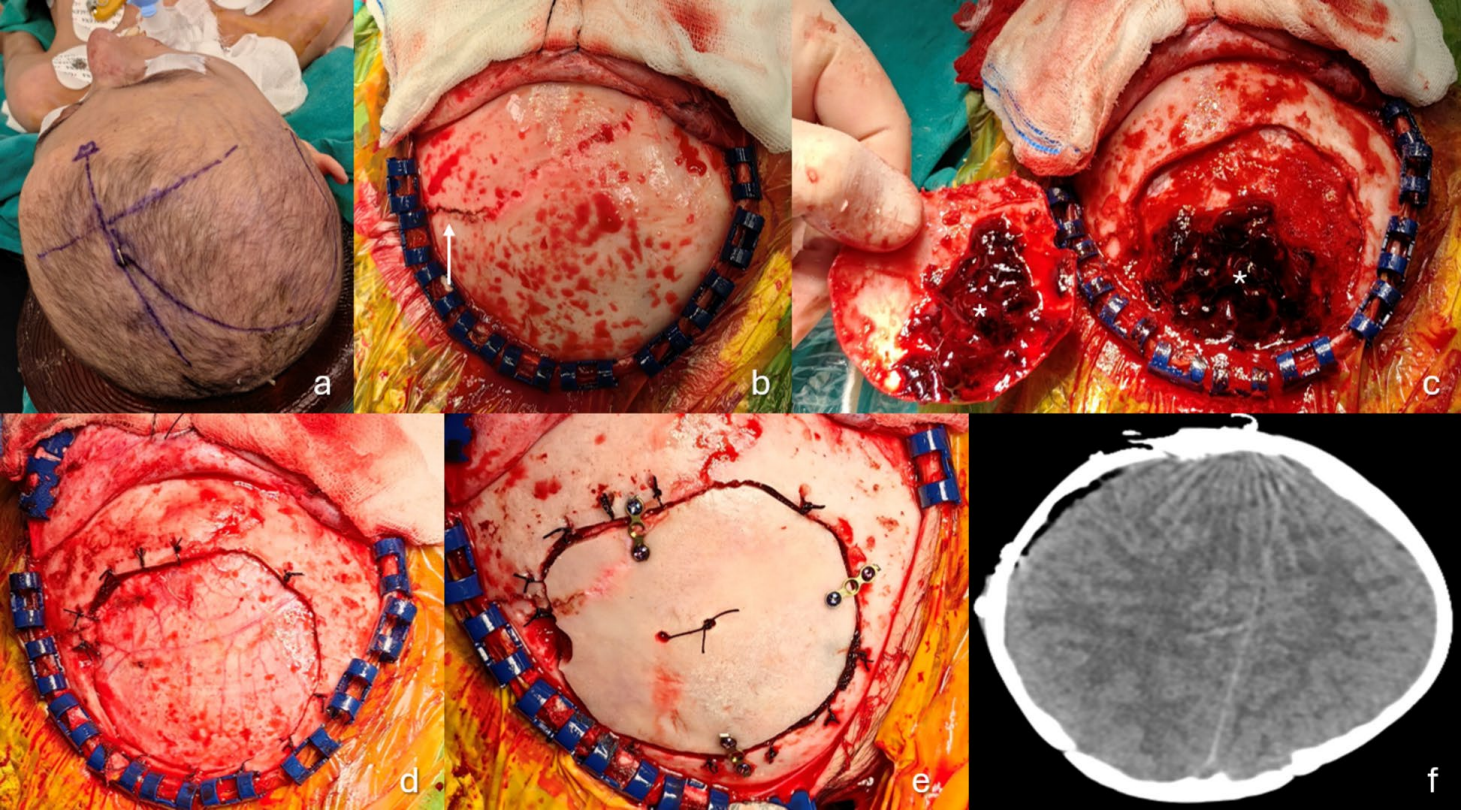
Preoperative positioning and incision planning (**a**), appearance of the skull vault during flap removal (**b**), epidural hematoma after craniotomy (**c**), appearance following hematoma evacuation (**d**), bone flap replacement (**e**), and postoperative noncontrast axial cranial CT (**f**). The patient’s head could not be turned due to ankylosis, allowing only up to a 15º rotation. Coronal suture diastasis with active bleeding from this area was observed (white arrow). An organized hematoma was encountered after craniotomy (asterisk). Standard surgical principles were applied throughout, and the procedure was completed without complications

The patient was positioned with silicone gel pads on all pressure points to protect the atrophied skin, and the head was placed on a doughnut-shaped pillow. Forced neck rotation was limited to 15° to avoid cervical spine injury (Fig. 2a). Following a standard craniotomy, significant bleeding was observed from multiple epidural veins associated with a diastasis of the coronal suture (Fig. 2b, c). Hemostasis was achieved through coagulation of the epidural veins, application of bone wax to the coronal diastasis, and repeated dural tenting sutures (Fig. 2d, e).

The procedure was completed without complications, and the patient was transferred to the pediatric intensive care unit with a patent tracheostomy for postoperative ventilation. Postoperative CT confirmed complete hematoma evacuation (Fig. 2f). She was moved to the ward after 5 days and discharged in stable condition on postoperative day 10. The tracheostomy was maintained for planned future orthodontic treatments.

## Discussion and conclusions

Patients with HGPS provide a unique opportunity to study the pathophysiological mechanisms of aging. Numerous studies on this condition have significantly advanced our understanding of aging biology by elucidating its pathogenesis and the underlying genetic and epigenetic mechanisms [1, 7]. According to the Progeria Research Foundation registry, approximately 140 children with HGPS are currently living worldwide [1]. Despite these insights, no curative treatment exists; currently, only the farnesyl transferase inhibitor lonafarnib has been approved by the Food and Drug Administration (FDA), reported to extend life expectancy by approximately 1.6 years [1, 7].

The sensitivity of elderly patients to trauma and the meticulous care required for surgical procedures are well recognized. However, trauma management in HGPS patients—who are extremely rare—is not well defined. In the literature, Mandera et al. [4] described a 10-year-old patient with progeria who developed EDH at two separate sites following mild trauma, highlighting the extreme fragility of this population. Another case involved a 7-year-old boy who presented with a severe EDH after a ground-level fall [5]. Our case is particularly significant as it represents the third reported instance of EDH in HGPS and is the first to provide a detailed overview of both anesthetic and operative management in a 15-year-old—the oldest survivor reported with this pathology. As demonstrated in Table 1, our patient’s advanced age correlated with more severe anatomical distortions compared to younger cases, necessitating a shift from standard intubation to emergency surgical airway management [4, 5].

**Table 1:** Comparative analysis of reported epidural hematoma cases

Feature	Mandera et al. (2003) [4]	Hansda et al. (2013) [5]	Present Case (2026)
Article Type	Case Report	Case Report	Case Report
Age/Gender	10 years/male	7 years/male	15 years/female
Trauma Mechanism	Fallen down	Minor fall while playing	Ground-level fall in playground
Admission GCS	10	N/A	12
Hematoma Location	Left temporal and posterior fossa	Right parietal	Right frontoparietal
Hematoma Thickness (cm)	2.5	N/A	2.2
Midline Shift (mm)	N/A	N/A	6
Surgical Nuance	Standart evacuation	Standart evacuation	Coronal suture diastasis; managed with bone wax and tenting
Anesthetic Nuance	N/A	Failed direct laryngoscopy; successful blind orotracheal intubation (4.5 mm tube)	Failed orotracheal/fiberoptic; Emergency percutaneous tracheostomy
Induction Agents	N/A	Deep inhalational anesthesia with spontaneous breathing	Propofol (2 mg/kg) / Fentanyl (1 mcg/kg)
Monitoring	N/A	Standard	Invasive Arterial BP (IBP) and Standard monitoring
Muscle Relaxants	N/A	N/A	None (Avoided due to severe cachexia)
Outcome	Good recovery	Good recovery	Good recovery; Post-op day 10 discharge

*N/A* Not available, *GCS* Glasgow Coma Scale

HGPS patients are highly susceptible to intracranial hemorrhage even after minor trauma due to progerin-induced vasculopathy. Progerin accumulation leads to a progressive loss of vascular smooth muscle cells and accelerated medial calcification. The progressive loss of vascular smooth muscle cells and the accelerated stiffening of the large arteries, as detailed in recent cardiovascular reviews of HGPS, underscore the extreme vulnerability of these patients to even low-energy cranial impacts [3]. This pathology, combined with potentially reduced intracranial compliance, may alter the brain's buffering capacity against expanding collections, leading to faster neurological deterioration compared to the general pediatric population [2]. While Nijs et al. [6] reviewed anesthetic considerations in HGPS, our experience confirms that for patients with advanced aging signs, clinicians should anticipate a "cannot intubate, cannot oxygenate" scenario. Standard orotracheal and even fiberoptic techniques (using a 2.8 mm device in our case) can fail due to progressive glottic sclerosis and hypertrophic arytenoids. In such cases, standard techniques can fail due to anatomic disruption, necessitating immediate transition to emergency surgical airway protocols to ensure rapid neurosurgical decompression.

In the presence of an acute traumatic EDH in HGPS, the choice of surgical intervention is dictated by the unpredictable and rapid expansion of the hematoma following a lucid interval, which poses an immediate life-threatening risk due to the underlying vascular fragility. While alternative decompression strategies have been discussed in broader neurosurgical contexts, they were deemed unsuitable for our patient due to the significant midline shift and the urgent requirement for definitive clot evacuation combined with direct hemostasis of the ruptured vessels at the coronal suture diastasis [8]. Neurosurgical management was further complicated by severe skin atrophy and ankylosis, requiring meticulous positioning with silicone gel pads and limiting neck rotation to 15° to prevent spinal injury. A unique finding was active bleeding from a coronal suture diastasis, controlled using bone wax and dural tenting sutures. Clinicians must maintain a high index of suspicion for intracranial injury in these patients, as pressure dynamics may not always correlate with findings in the general population. In conclusion, prompt decision-making for tracheostomy, avoidance of anesthetic delays, and meticulous execution of surgical procedures are essential for favorable outcomes in this high-risk population.

## Data Availability

The data supporting the findings of this study are available from the corresponding author upon reasonable request.

## Abbreviations

HGPS: Hutchinson–Gilford progeria syndrome
EDH: Epidural hematomas
CT: Computerized tomography
GCS: Glasgow Coma Scale
BMI: Body Mass Index
INR: International Normalized Ratio
ICP: Intracranial pressure
IBP: Intraarterial blood pressure
FDA: Food and Drug Administration
